# Supplementary material for: NOTCH signaling specifies arterial-type definitive hemogenic endothelium from human pluripotent stem cells
Source: Nat Commun. 2018 May 8;9:1828. doi: 10.1038/s41467-018-04134-7 (PMC5940870; doi:10.1038/s41467-018-04134-7)
Supplement: Supplementary file 1 — Supplementary Information [file 41467_2018_4134_MOESM1_ESM.pdf]

Supplemental Materials for

Uenishi et al.

NOTCH Signaling Specifies Arterial-Type Definitive Hemogenic Endothelium from Human Pluripotent Stem Cells Ueneshi et al

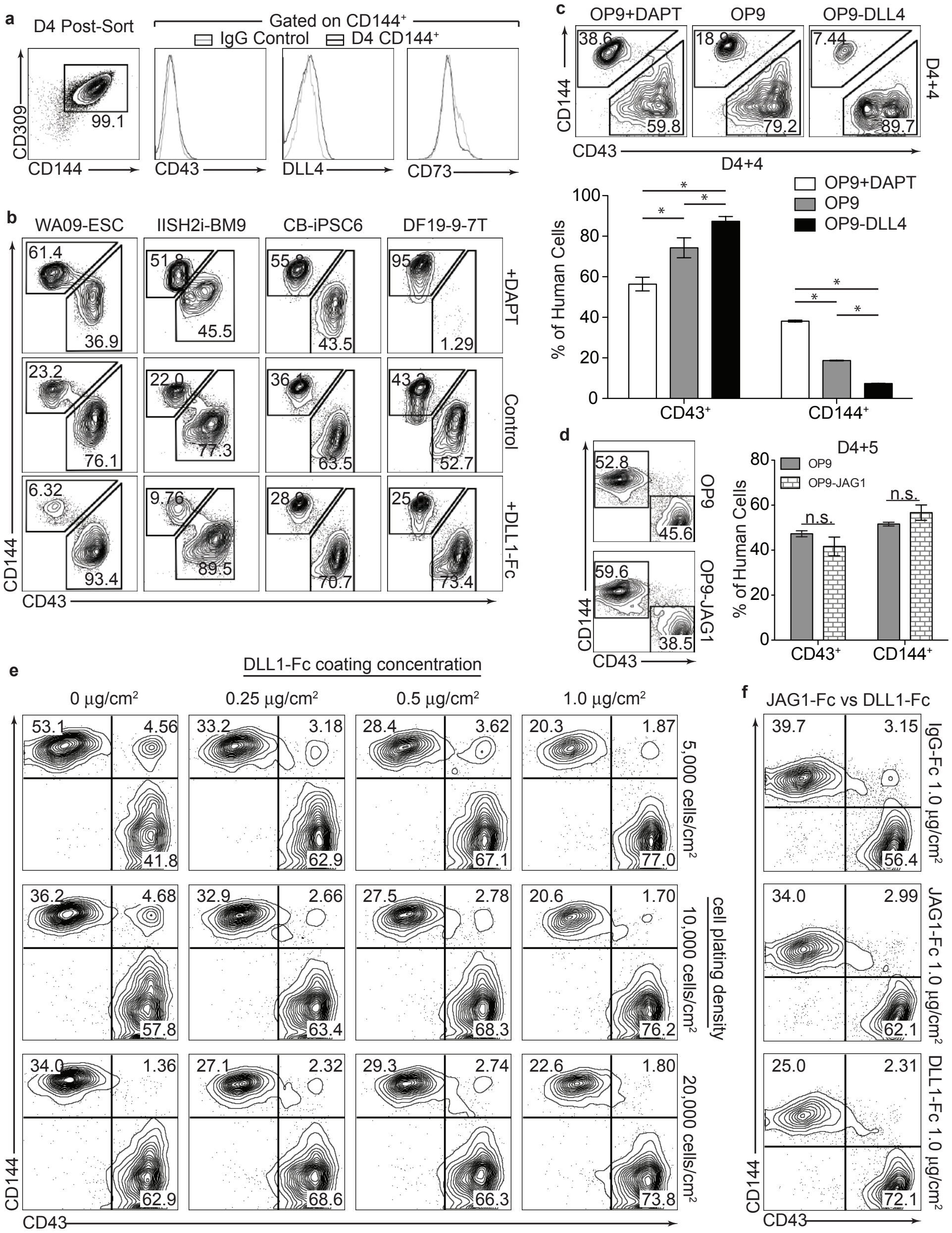

Supplementary Figure 1. Determining the effect of NOTCH signaling on EHT. (a) Phenotype of day 4 CD144+ cells. (b) Effect of NOTCH inhibition and activation on hematopoiesis from D4 HE cells generated from WA09 ESCs, and iPSCs derived from bone marrow hematopoietic cells (IISH2i-BM9), cord blood (CB-iPSC6) and dermal fibroblasts (DF19-9-7T). The NOTCH effects are consistent across different hPSC lines. (c) Evaluation of EHT from D4 HE cultured on OP9, OP9-DLL4 or in presence of DAPT. NOTCH activation had similar effects on hematopoiesis whether in stroma/serum or stroma-/serum-free conditions. (d) Evaluation of EHT from D4 HE cultured on OP9 versus OP9-JAG1. OP9-JAG1 had little effect on EHT, unlike OP9-DLL4. Results in (c) and (d) are mean ± SEM for at least 3 independent experiments; 2-way ANOVA Bonferroni post-hoc test, *p<.05, **p<.01, ***p<.001. (e) Measuring the effect of increasing concentrations of DLL1-Fc with increasing cell density of D4 HE cells. (e) Effect of JAG1-Fc on hematopoiesis from day 4 HE. Representative histograms and contour plots from 3 independent experiments are shown.

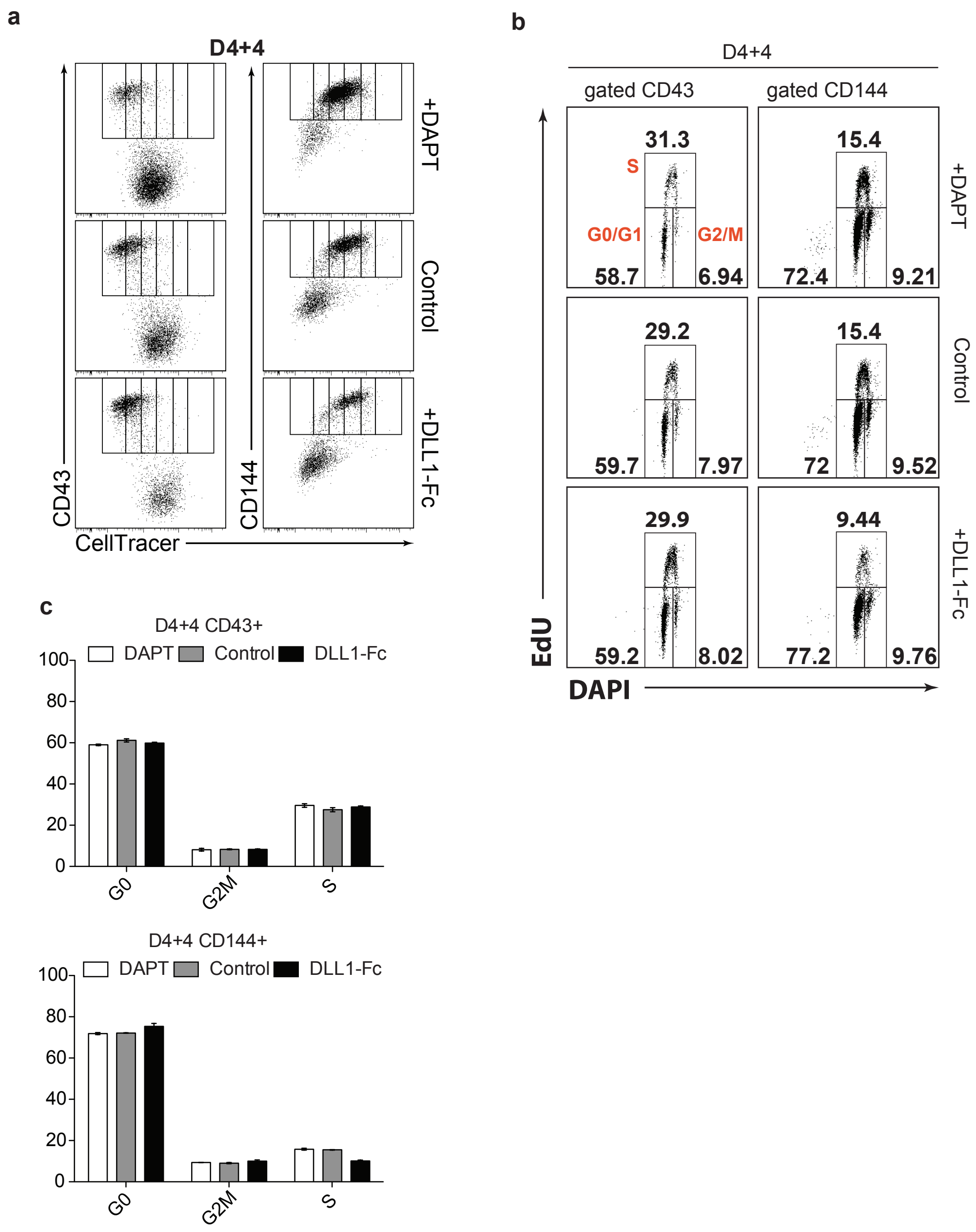

Supplementary Figure 2. Effect of NOTCH signaling on proliferation and cycling of D4+4 cells. (a) Representative flow cytometric cell proliferation analysis representing at least 3 independent experiments conducted with CellTracer. Generation gates were determined by concatenating D4 to D4+4 results and utilizing FlowJo's proliferation assay. (b) Representative dot plots show flow cytometric analysis of cell cycle using EdU and DAPI staining on D4+4 cells. (c) Bar graphs reveal no significant changes in cell cycling phases between each condition on D4+4. Results are mean ± s.e.m. n=3.

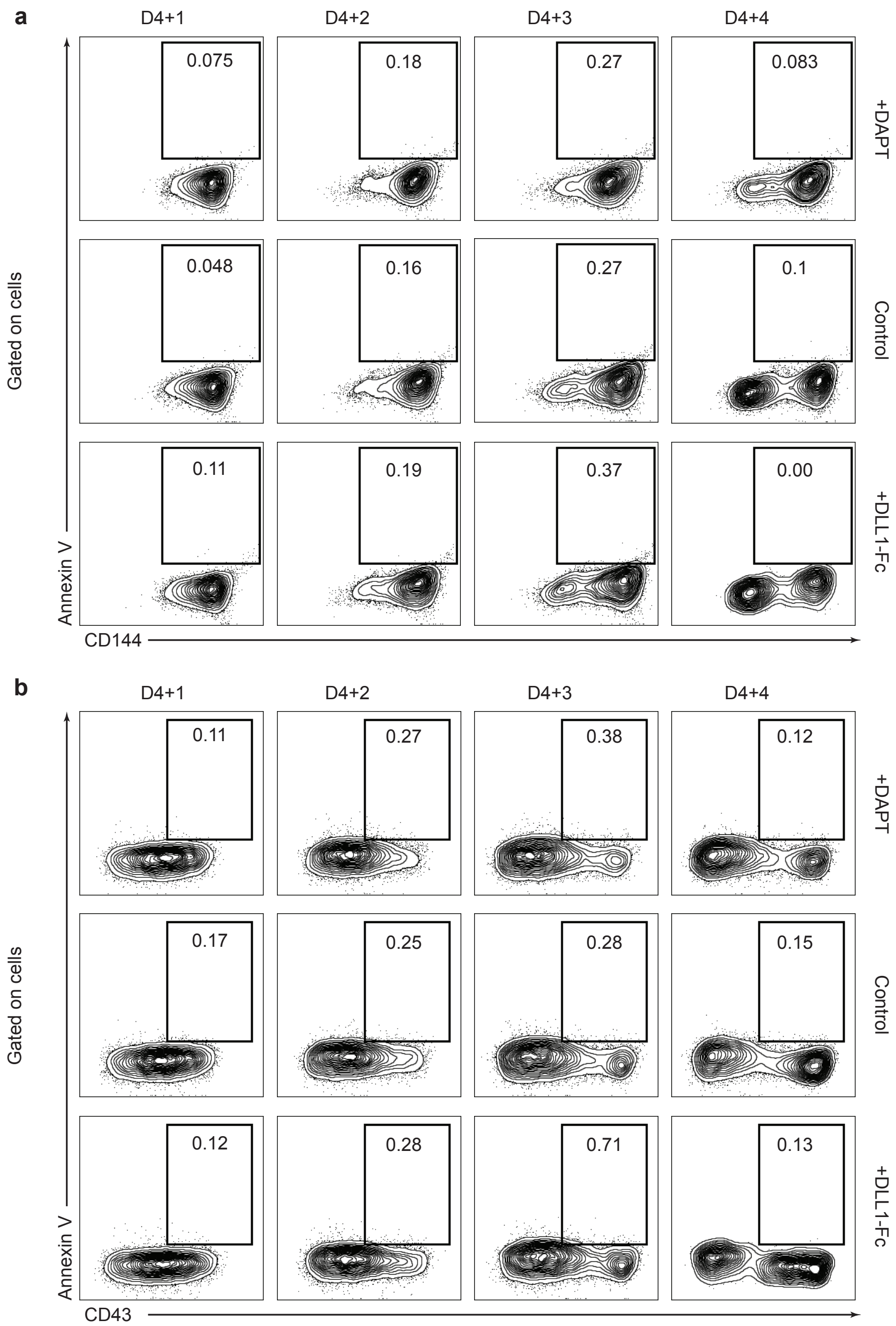

Supplementary Figure 3. Flow cytometry of Annexin V to determine apoptosis during secondary culture of D4 HE cells in the presence of DAPT or DLL1-Fc. Flow cytometry showing the percent of apoptotic cells via Annexin V staining in the (a) endothelial and (b) hematopoietic populations. Lack of significant differences in apoptotic cells in different conditions provides evidence that NOTCH signaling does not affect cell survival following EHT. Representative contour plots from 3 independent experiments are shown.

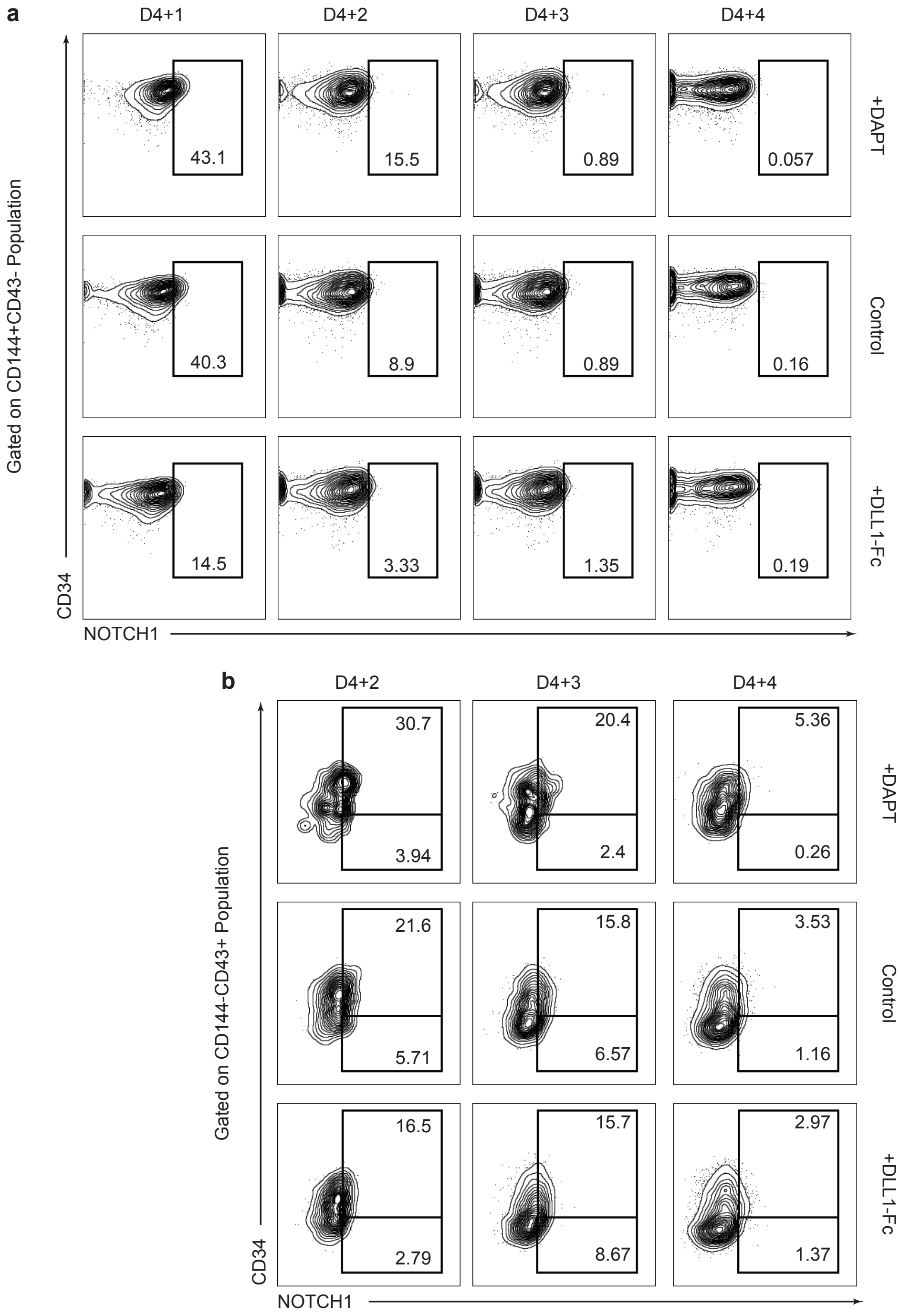

Supplementary Figure 4. NOTCH1 expression in $CD34^{+}$ hematoendothelial populations during secondary culture of D4 HE cells in the presence of DAPT or DLL1-Fc. (a) Expression of NOTCH1 on endothelial cells following secondary culture of D4 HE cells. $CD144^{+}CD43^{-}$ endothelial cells have decreased NOTCH1 expression from D4+1 to D4+4 and (b) Expression of NOTCH1 on hematopoietic cells following secondary culture of D4 HE cells. $CD144^{-}CD34^{+}CD43^{+}$ hematopoietic progenitors have increased NOTCH1 expression D4+2 to D4+4 as compared to $CD34^{-}CD43^{+}$ cells.

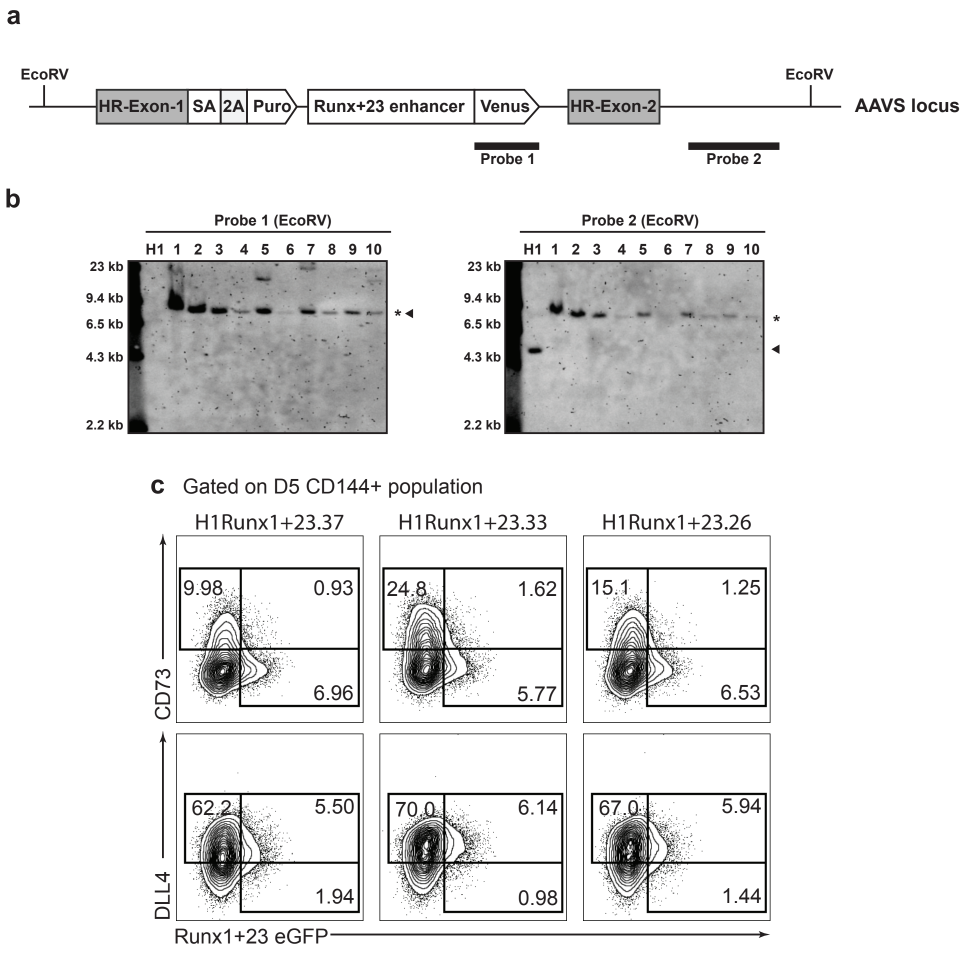

Supplementary Figure 5. Generation of RUNX1+23-eGFP reporter WA01 hESC line.
a) Schematic diagram of the construct used for the targeting of RUNX1+23-eGFP reporter into AAVS locus. Donor plasmid was integrated into the cleavage location of the Zinc Finger-Nuclease pair. b) Southern blot analysis of the WA01 cells targeted with the donor plasmid containing RUNX1+23-eGFP construct. Blot shows EcoRV digested genomic DNA hybridized with DIC-labeled 5' internal probe 1 (wt=no band, targeted=8.1kb) and 3'external probe 2 (wt=5.4kb, targeted=8.8kb). Filled arrow = wild type; Asterisk = targeted c) D5 flow of 3 different RUNX1+23 reporter hESC lines reveals that all eGFP+ cells are DLL4^{+}CD73^{-}.

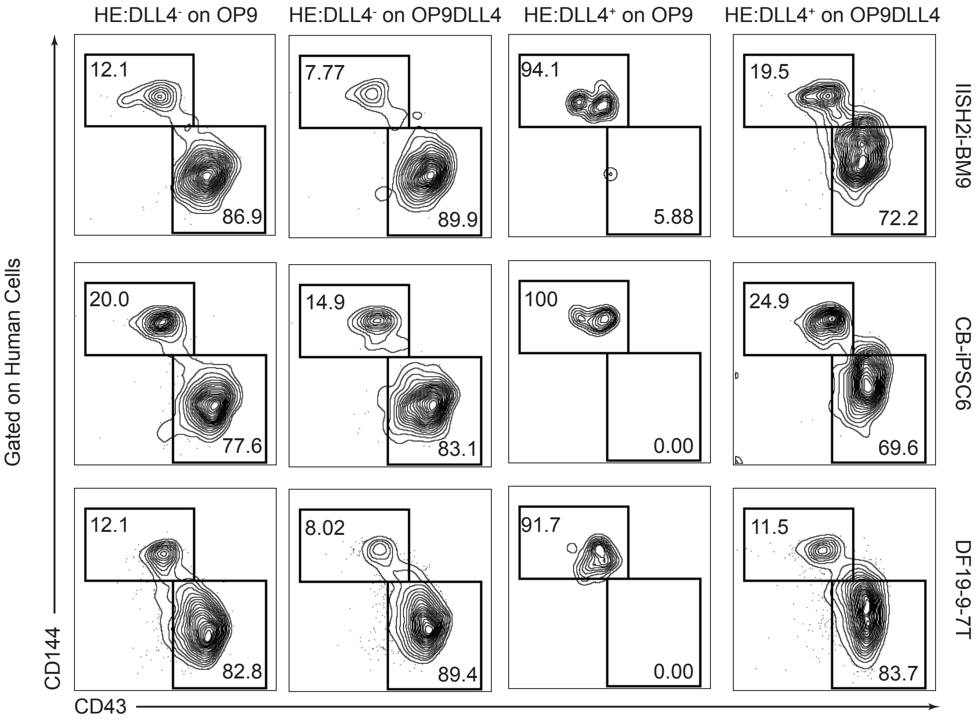

Supplementary Figure 6. D5 HE subsets derived from different hiPSC lines have the same response to OP9 and OP9-DLL4. Bone marrow IISH2iBM9, cord blood CB-iPSC6 or fibroblast-derived DF19-9-7T iPSCs were differentiated for 5 days in defined conditions. D5 HE:DLL4$^-$ and D5 HE:DLL4$^+$ were sorted and cultured on OP9 or OP9-DLL4 for 4 days. D5+4 flow plots of D5 HE cells demonstrate that D5 HE:DLL4$^+$ cells show hemogenic activity from only when cultured on OP9-DLL4. One experiment for each cell line is shown.

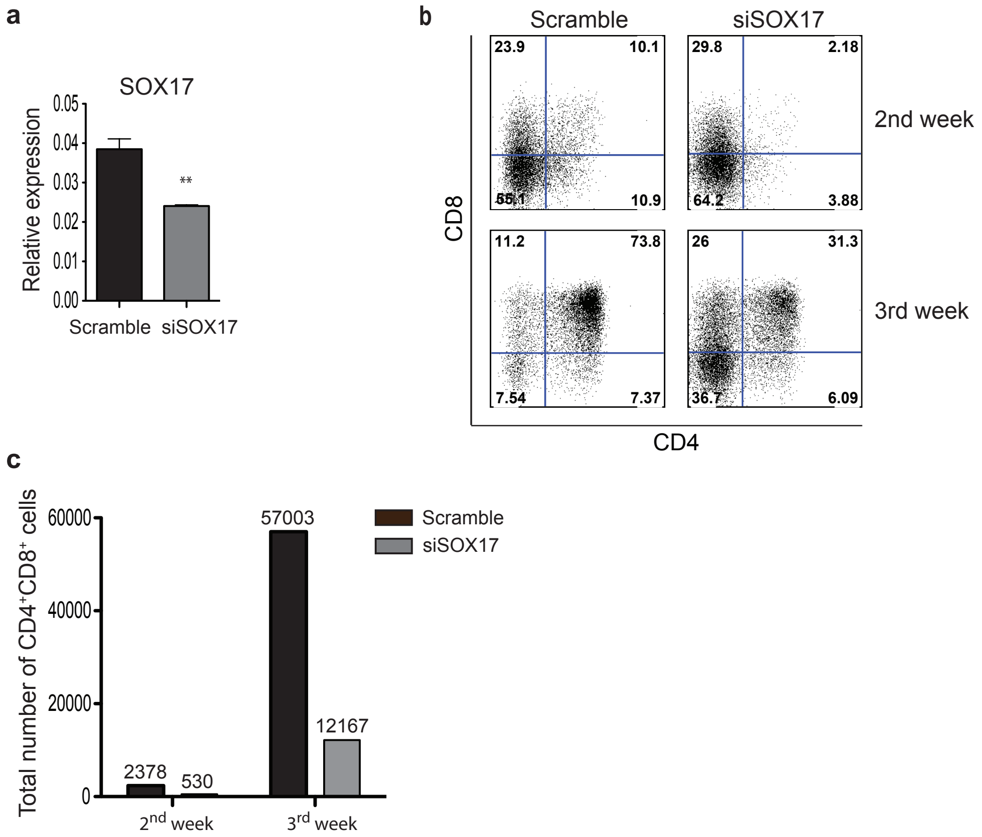

Supplementary Figure 7. Knockdown of *SOX17* arterial gene with siRNA impairs lymphoid development from hESCs. a) hESC differentiation cultures were transfected with SOX17 or scramble siRNA on D3 of differentiation. Graph shows qPCR analysis of SOX17 mRNA expression 48 hours after transfection (D5 of differentiation). Results are mean ± SEM for at least 3 independent experiments; student's t-test, *p<.05, **p<.01, ***p<.001. b) and c) shows T cell production by HPs collected on D8 of differentiation from cultures treated with SOX17 and scramble siRNA on D3 of differentiation. Representative dot plots (b) and total numbers of T cells produced from 10^4 HPs (c) after 2 and 3 weeks of culture in T cell conditions are shown.

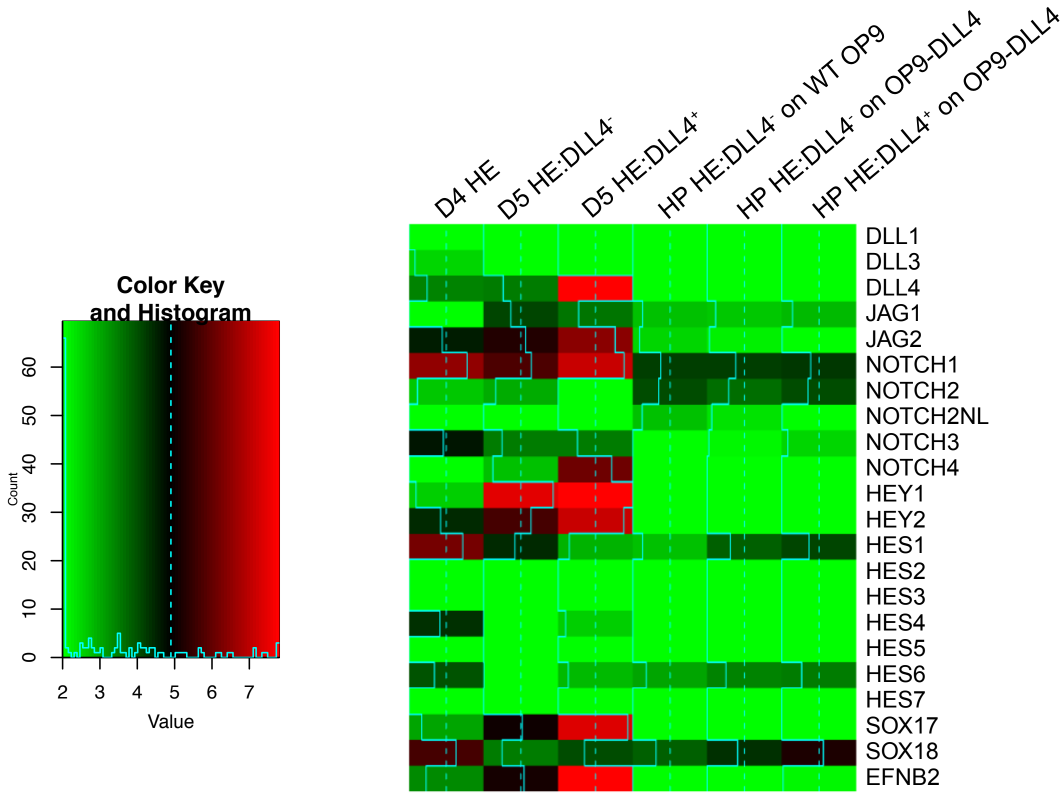

Supplementary Figure 8. Expression of arterial and NOTCH signaling-associated genes in HE and HPs. Heat map demonstrates expression of NOTCH signaling associated and arterial genes in immature D4 HE, D5 HE:DLL4$^+$ and HE:DLL4$^-$, and hematopoietic progenitors CD34$^+$CD45$^+$ generated from D5 HE:DLL4$^+$ and HE:DLL4$^-$ on wild type OP9 or OP9-DLL4 as depicted in Figure 6a. Log2-transformed Transcripts Per Million (log2(TPM)) are used for color mapping. The color gradient is set to reflect highly expressed genes as red, non-expressed genes as green and genes expressed at 30 tpm as black.

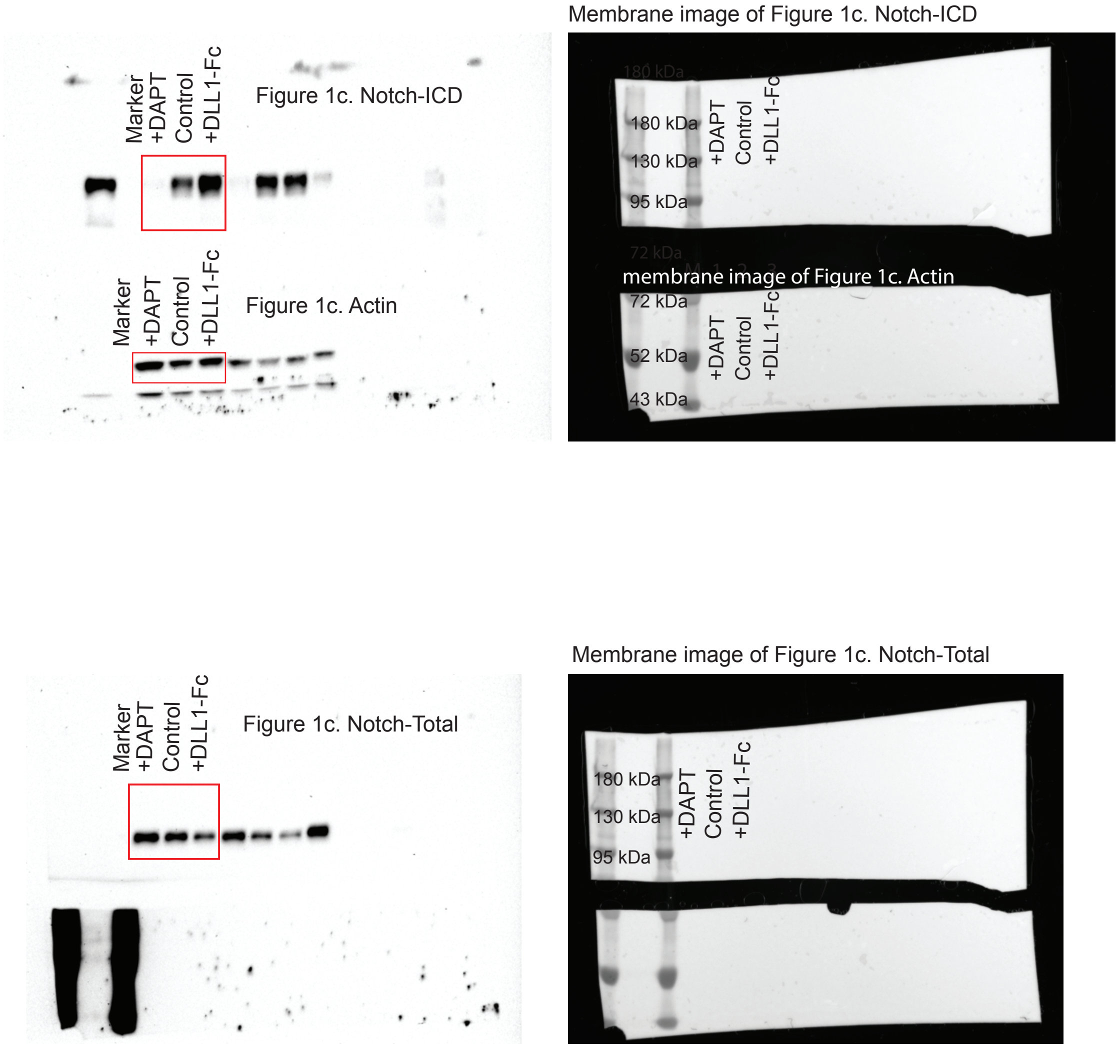

Supplementary Figure 9. Uncropped image of western blot presented in Figure 1c.

Supplementary Table 1. Fluorescent Reagents Used in This Study

Reagent	Fluorescence	Source	Cat No
7-AAD	488/647	Cayman Chemicals	11397
Annexin V	PE	BD Biosceinces	556421
CellTracer	405/450	ThermoFisher	C34557
Ghost Dye Red 780	633/780	Tonbo Bio	13-0865
Ghost Dye Violet 510	405/510	Tonbo Bio	13-0870

Supplementary Table 2. Antibodies Used in This Study

Antigen	Conjugate	Source	Clone	Cat No.	Dilution
CD4	APC	BD Bioseceinces	RPA-T4	555349	1:50
CD5	PE-Vio770	Miltenyi Biotec	130-111-109	REA782	1:50
CD7	FITC	Miltenyi Biotec	130-105-844	CD7-6B7	1:50
CD8	PE	BD Bioseceinces	HIT8a	555635	1:50
CD31	FITC	BD Bioseceinces	WM59	555445	1:50
CD31	PE	BD Bioseceinces	WM59	555446	1:50
CD31	MicroBeads	Miltenyi Biotec	N/A	130-091-935	1:50
CD34	FITC	BD Bioseceinces	8G12	555821	1:50
CD34	PE-Vio770	Miltenyi Biotec	24D2	130-100-844	1:50
CD41a	PE	BD Bioseceinces	HIP8	555467	1:50
CD41a	APC	BD Bioseceinces	HIP8	559777	1:50
CD41a	PE-Cy5	BD Bioseceinces	HIP8	559768	1:50
CD41a	PE-Cy7	BD Bioseceinces	HIP8	561424	1:50
CD41a	FITC	BD Bioseceinces	HIP8	555466	1:50
CD41a	PerCP-Cy5.5	BD Bioseceinces	HIP8	340931	1:50
CD41a	PE	Miltenyi Biotec	REA386	130-105-612	1:50
CD41a	APC-Vio770	Miltenyi Biotec	REA386	130-105-563	1:50
CD43	PE	BD Bioseceinces	1G10	560199	1:50
CD43	APC	BD Bioseceinces	1G10	560198	1:50
CD43	BV421	BD Bioseceinces	1G10	562916	1:50
CD43	BV510	BD Bioseceinces	1G10	563377	1:50
CD43	PE	Miltenyi Biotec	DF-T1	130-097-362	1:50
CD43	APC	Miltenyi Biotec	DF-T1	130-097-367	1:50
CD43	APC-Vio770	Miltenyi Biotec	DF-T1	130-101-174	1:50
CD43	VioBlue	Miltenyi Biotec	DF-T1	130-097-373	1:50
CD43	purified	BD Bioseceinces	1G10	551457	1:50
CD45	PE	BD Bioseceinces	HI30	555483	1:50
CD45	APC	BD Bioseceinces	HI30	555485	1:50
CD45	BV421	BD Bioseceinces	HI30	563879	1:50
CD45	PE	Miltenyi Biotec	130-080-201	5B1	1:50
CD45	PE-Vio770	Miltenyi Biotec	130-096-616	5B1	1:50
CD45	APC	Miltenyi Biotec	130-091-230	5B1	1:50
CD45	APC-Vio770	Miltenyi Biotec	130-096-609	5B1	1:50
CD73	PE	BD Bioseceinces	AD2	550257	1:50
CD73	PE-Cy7	BD Bioseceinces	AD2	561258	1:50
CD73	Purified	BD Bioseceinces	AD2	550256	1:50
CD73	APC	BD Bioseceinces	AD2	560847	1:50
CD73	FITC	BD Bioseceinces	AD2	561254	1:50

Antigen	Conjugate	Source	Clone	Cat No.	Dilution
CD73	BV421	BD Bioesceinces	AD2	562430	1:50
CD144	PE	BD Bioesceinces	55-7H1	560410	1:50
CD144	FITC	BD Bioesceinces	55-7H1	560411	1:50
CD144	PerCP-Cy5.5	BD Bioesceinces	55-7H1	561566	1:50
CD144	PE-Vio770	Miltenyi Biotec	REA199	130-100-720	1:50
CD144	VioBlue	Miltenyi Biotec	REA199	130-100-724	1:50
CD144	purified	eBioscience	BV13	14-1441	1:50
CD184	PE	BD Bioesceinces	12G5	555974	1:50
CD184	PerCP-Cy5.5	BD Bioesceinces	12G5	560670	1:50
CD235a	PE	BD Bioesceinces	GA-R2 (HIR2)	555570	1:200
CD235a	FITC	BD Bioesceinces	GA-R2 (HIR2)	559943	1:200
CD235a	APC	BD Bioesceinces	GA-R2 (HIR2)	551336	1:200
CD309	PE	BD Bioesceinces	89106	560494	1:50
CD309	Alexa Fluor® 647	BD Bioesceinces	89106	560495	1:50
CD309	APC	Miltenyi Biotec	ES8-20E6	130-098-910	1:50
Actin	purified	Santa Cruz	C-2	SC-8432	1:2000
Mouse IgG	Alexa Fluor® 488	Life Technologies	polyclonal	A11001	1:50
Mouse IgG	HRP	Santa Cruz	polyclonal	SC-2005	1:1000
DLL4	PE	Miltenyi Biotec	MHD4-46	130-096-567	1:50
DLL4	PE-Vio770	Miltenyi Biotec	MHD4-46	130-101-563	1:50
DLL4	PE	R&D Systems	447506	FAB1506P	1:50
NOTCH1	APC	R&D Systems	527425	FAB5317A	1:50
NOTCH1	purified	Cell Signaling Tech	C37C7	3439	1:50
NOTCH1-ICD	purified	Cell Signaling Tech	D3B8	4147	1:100
Rabbit IgG	Alexa Fluor® 594	Life Technologies	polyclonal	A11012	1:50
Rabbit IgG	HRP	Santa Cruz	polyclonal	SC-2004	1:1000

Supplementary Table 3. Primers used for qRT-PCR in This Study

Gene	Forward Sequence	Reverse Sequence
CXCR4	TCAGTGGCTGACCTCCTCTT	CTTGGCCTTTGACTGTTGGT
DLL4	CAGTGGGCAGCGAAGCTACA	ACAGGCAGTGGTAGCCATCCTC
EPHB2	CTCCTCAACTGTGCCAAACCA	GGTTATCCAGGCCCTCCAAA
GATA2	CCCTAAGCAGCGCAGCAA	TGACTTCTCCTGCATGCACT
HAND1	GCCTACCTGATGGACGTGCT	GCCGGTGCGTCCTTTAATCC
HBA2	CGGTCAACTTCAAGCTCCTAA	GCCCACTCAGACTTTATT
HBB	GGCACCTTTGCCACACTG	CACTGGTGGGGTGAATTCTT
HBE1	GCCTGTGGAGCAAGATGAAT	GCGGGCTTGAGGTTGT
HBG1	CTTCAAGCTCCTGGGAAATGT	GCAGAATAAAGCCTATCCTTGAAAG
HBZ1	CGGTGAAGAGCATCGACG	GGATACGACCGATAGGAACTTGT
HES1	TACCCCAGCCAGTGTCAAC	TCAGCTGGCTCAGACTTTCA
HEY2	TTCAAGGCAGCTCGGTAACTGAC	CATACTGATGCACTGCTGGATGG
MYB	ACGGTCCGAAACGTTGGTCTG	CCCCAGTCTCTTGTGTGCCTGG
NOTCH1	CAATGTGGATGCCGCAGTTGTG	CAGCACCTTGGCGGTCTCGTA
NR2F2	TGGTTCCAAACCAGTTTATTCTGT	AAGTGCGTTTCCATCATCTTTGAG
RPL13a	CCTGGAGGAGAAGAGGAAAGAGA	TTGAGGACCTCTGTGTATTTGTCAA
SOX17	GCCAAGGGCGAGTCCCGTA	GCATCTTGCTCAACTCGGCGTTGTGCA
