# Supplementary material for: NOTCH signaling specifies arterial-type definitive hemogenic endothelium from human pluripotent stem cells
Source: Nat Commun. 2018 May 8;9:1828. doi: 10.1038/s41467-018-04134-7 (PMC5940870; doi:10.1038/s41467-018-04134-7)
Supplement: Supplementary file 3 — Description of Additional Supplementary Files [file 41467_2018_4134_MOESM3_ESM.docx]

**Description of Additional Supplementary Files**

File Name: Supplementary Data 1

Description: Differentially expressed genes in CD34+CD43+CD45+CD235a/CD41a- hematopoietic progenitors generated from DLL4- HE on OP9 and OP9-DLL4 and DLL4+ HE on OP9-DLL4.
